# Supplementary material for: Information nudges for influenza vaccination: Evidence from a large-scale cluster-randomized controlled trial in Finland
Source: PLoS Med. 2022 Feb 9;19(2):e1003919. doi: 10.1371/journal.pmed.1003919 (PMC8870595; doi:10.1371/journal.pmed.1003919)
Supplement: S1 Appendix — Table A. Average treatment effects estimated using linear probability models, logit models, and generalized mixed effects regression with random effects. Table B. Average treatment effects with and without control variables. Table C. The effect of reminders on influenza vaccine coverage by prior immunization history—Random effects linear model. Table D. Cross-vaccination spillovers to other age-appropriate vaccines. Fig A. Minimal detectable effect sizes (with α = 0.05 and 0.80) for treatment comparisons by intracluster correlation coefficients in the Western region. Fig B. Minimal detectable effect sizes (with α = 0.05 and 0.80) for the joint effect of any type of reminder by intracluster correlation coefficients in the Western region. Fig C. Minimal detectable effect sizes (with α = 0.05 and 0.80) for the treatment comparison by intracluster correlation coefficients in the Southern region. (DOCX) [file pmed.1003919.s002.docx]

*ONLINE APPENDIX for*

Information nudges for influenza vaccination:

Evidence from a large-scale cluster-randomized controlled trial in Finland

Lauri Sääksvuori^1,2,3*^, Cornelia Betsch^4,5^, Hanna Nohynek^6^,

Heini Salo^6^, Jonas Sivelä^6^, Robert Böhm^7,8^

^1^ Tampere University, Department of Health Sciences, Faculty of Social Sciences, Tampere, Finland

^2^ University of Turku, INVEST Research Flagship Center, Turku, Finland

^3^ Finnish Institute for Health and Welfare, Centre for Health and Social Economics, Helsinki, Finland

^4^ University of Erfurt, Media and Communication Science and Center for Empirical Research in Economics and Behavioral Sciences, Erfurt, Germany

^5^ Hamburg University, Bernhard Nocht Institute for Tropical Medicine, Hamburg, Germany

^6^ Finnish Institute for Health and Welfare, Infectious Disease Control and Vaccinations, Department of Health Security, Helsinki, Finland

^7^ University of Vienna, Faculty of Psychology, Vienna, Austria

^8^ University of Copenhagen, Department of Psychology and Copenhagen Center for Social Data Science (SODAS), Copenhagen, Denmark

[lauri.saaksvuori@thl.fi](mailto:lauri.saaksvuori@thl.fi)

**Appendix A: Original letters and English translation**

**A1: Finnish (individual-benefit reminder)**

KUTSU

16.11.2018

**Tervetuloa influenssarokotukseen!**

Kutsumme kaikki Rannikko-Pohjanmaan alueen (Maalahti, Korsnäs, Närpiö, Kaskinen ja Kristiinankaupunki) 65 vuotta täyttäneet maksuttomaan influenssarokotukseen.

Influenssa on yleinen ja vakava tauti ikääntyneillä. Rokote on paras keino suojautua influenssalta. Se suojaa myös influenssan jälkitaudeilta, esimerkiksi keuhkokuumeelta. Rokote on hyvä ottaa joka syksy, sillä rokotuksen suojateho kestää noin vuoden verran. Influenssavirus muuntuu jatkuvasti, ja siksi aiemmin otettu rokote ei suojaa riittävästi enää seuraavana vuonna.

**Voit tulla influenssarokotukseen ilman ajanvarausta alla olevina aikoina**

**Kristiinankaupungin neuvola (Lapväärtintie 10)**

- maanantai 29.10. klo 13–17
- maanantai 5.11. klo 13–17
- maanantai 26.11. klo 13–17
- maanantai 10.12. klo 13–17

**Siipyyn neuvola (Långvikintie 16)**

- keskiviikko 31.10. klo 12–15
- keskiviikko 14.11. klo 12–15

**Voit myös varata rokotusajan arkisin klo 8.30–9.30 numerosta 06 221 8480**

Ota Kela-kortti mukaan, kun tulet rokotukseen. Rokotusta varten kannattaa pukeutua niin, että käsivarsi on helppo paljastaa.

**Jos olet kotihoidon asiakas tai asut hoivakodissa**, saat influenssarokotuksen näiden palvelujesi yhteydessä. Sinun ei tarvitse varata aikaa eikä lähteä erikseen rokotukseen.

**Tervetuloa!

Peter Riddar,** ylilääkäri

Lisätietoa: www.kausi-influenssa.fi

Osoitelähde: Väestötietojärjestelmä, Väestörekisterikeskus, PL 123, 00531 Helsinki

Kutsut toteutetaan yhdessä Terveyden ja hyvinvoinnin laitoksen kanssa.

**A2: Swedish (individual-benefit reminder)**

KALLELSE

16.11.2018

**Välkommen på influensavaccination!**

Vi bjuder in alla som fyllt 65 år i Kust-Österbotten (Malax, Korsnäs, Närpes, Kaskö och Kristinestad) till en avgiftsfri influensavaccinering.

Influensa är en allmän och allvarlig sjukdom bland äldre. Vaccinet är det bästa sättet att skydda sig mot influensa. Det skyddar även mot följdsjukdomar, som till exempel lunginflammation. Det är bra att vaccinera sig varje höst eftersom skyddseffekten håller cirka ett år. Influensaviruset förändras hela tiden, och därför skyddar ett vaccin som man tagit tidigare inte längre tillräckligt bra följande år.

**Du kan komma och vaccinera följande tider utan tidsbeställning**

**Kristinestads rådgivning (Lappfjärdsvägen 10)**

- måndag 29.10. kl. 13–17
- måndag 5.11. kl. 13–17
- måndag 26.11. kl. 13–17
- måndag 10.12. kl. 13–17

**Sideby rådgivning (Långvikvägen 16)**

- onsdag 31.10 kl. 12–15
- onsdag 14.11 kl. 12–15

**Du kan även boka tid för vaccinationen under vardagar kl. 8.30–9.30 på numret 06 221 8480**

Tag med ditt FPA-kort till vaccineringen. För vaccineringen är det bra att välja kläder så att du enkelt kan kavla upp ärmen.

**Om du är kund hos hemtjänsten eller om du bor på servicehem** får du influensavaccinet i samband med dessa tjänster. Du behöver inte boka tid eller komma och vaccinera dig separat.

**Välkommen!**

**Peter Riddar,** överläkare

Ytterligare upplysningar: www.sasongsinfluensa.fi

Adresskälla: Befolkningsdatasystemet, Befolkningsregistercentralen, PB 123, 00531 Helsingfors

Kallelsen genomförs i samarbete med Institutet för hälsa och välfärd.

**A3: English Translation (individual-benefit reminder)**

**Invitation to Influenza Vaccination!**

We would like to invite all citizens aged 65 and above living in Coastal Ostrobothnia (Maalahti, Korsnäs, Närpiö, Kaskinen and Kristiinankaupunki) for free influenza immunization.

Seasonal influenza is a common and serious disease in the age group of 65 and above. Influenza vaccination is the best way to protect you against the disease. Influenza vaccine will protect you also against many secondary diseases associated with seasonal influenza such as pneumonia. It is recommended to take an influenza vaccine every autumn as the protective effect of these vaccines last for about a year. Influenza viruses continuously change and previously taken vaccines may not provide protection in the following years.

**You may receive your influenza vaccine without appointment at following dates and times**

**Kristiinankaupunki – Children’s health clinic (Address: Lapväärtintie 10)**

- **Monday 29.10. from 1 p.m. to 5 p.m.**
- **Monday 05.11. from 1 p.m. to 5 p.m.**
- **Monday 26.11. from 1 p.m. to 5 p.m.**
- **Monday 10.12. from 1 p.m. to 5 p.m.**

**Siipyy – Children’s health clinic (Address: Långvikintie 16)**

- **Wednesday 31.10. from noon to 3 p.m.**
- **Wednesday 14.11. from noon to 3 p.m.**

**You may also book an appointment for the vaccine administration on weekdays by calling 06 221 8480**

Please bring you social security card with you. We recommend wearing clothes that enable injection of *a vaccine into the shoulder.*

**Please notice that if you receive medical home care or live in a nursing home,** you may receive an influenza vaccine directly through your care givers. You do not have to book an appointment and travel to receive your vaccine.

**Welcome**

**Peter Riddar,** Chief physician

For additional information please contact: [www.kausi-influenssa.fi](http://www.kausi-influenssa.fi)

Your mail address was extracted from the Population Register, Population Register Center, P.0. Box 123, 00531 Helsinki

This invitation has been prepared in cooperation with the Finnish Institute for Health and Welfare.

**A4: English Translation (individual- and social-benefit reminder)**

**Invitation to Influenza Vaccination!**

We would like to invite all citizens aged 65 and above living in Coastal Ostrobothnia (Maalahti, Korsnäs, Närpiö, Kaskinen and Kristiinankaupunki) for free influenza immunization.

Seasonal influenza is a common and serious disease in the age group of 65 and above. Influenza vaccination is the best way to protect you against the disease. Influenza vaccine will protect you also against many secondary diseases associated with seasonal influenza such as pneumonia. It is recommended to take an influenza vaccine every autumn as the protective effect of these vaccines last for about a year. Influenza viruses continuously change and previously taken vaccines may not provide protection in the following years.

Your decision to vaccinate does not only protect you but others as well. Your vaccination may protect small children whose immune system is still developing. You will be able to protect your loved ones who are unable to get vaccinated. Your vaccination may prevent the spread of influenza viruses. Thus, the whole society benefits from your decision to vaccinate.

**You may receive your influenza vaccine without appointment at following dates and times**

**Kristiinankaupunki – Children’s health clinic (Address: Lapväärtintie 10)**

- **Monday 29.10. from 1 p.m. to 5 p.m.**
- **Monday 05.11. from 1 p.m. to 5 p.m.**
- **Monday 26.11. from 1 p.m. to 5 p.m.**
- **Monday 10.12. from 1 p.m. to 5 p.m.**

**Siipyy – Children’s health clinic (Address: Långvikintie 16)**

- **Wednesday 31.10. from noon to 3 p.m.**
- **Wednesday 14.11. from noon to 3 p.m.**

**You may also book an appointment for the vaccine administration on weekdays by calling 06 221 8480**

Please bring you social security card with you. We recommend wearing clothes that enable injection of *a vaccine into the shoulder.*

**Please notice that if you receive medical home care or live in a nursing home,** you may receive an influenza vaccine directly through your care givers. You do not have to book an appointment and travel to receive your vaccine.

**Welcome**

**Peter Riddar,** Chief physician

For additional information please contact: [www.kausi-influenssa.fi](http://www.kausi-influenssa.fi)

Your mail address was extracted from the Population Register, Population Register Center, P.0. Box 123, 00531 Helsinki

This invitation has been prepared in cooperation with the Finnish Institute for Health and Welfare.

**Appendix B: Randomization script**

* Data management after receiving the address data

import excel “\\helfs01.thl.fi\cData\Rokoteviesti_RCT\Tulokset 2018-09-28.xlsx”, sheet(“Tulostiedot”) firstrow

* KEEP only Kaskinen

keep if Kunnannimi==”Kaskinen”

*Create unique identifiers by apartment

sort Asuinpaikantunnus

egen running_apartment = group(Asuinpaikantunnus)

bys Asuinpaikantunnus: gen n_of_persons = _N

set seed 21042403

gen random_number =uniform()

bysort Asuinpaikantunnus: replace random_number = random_number[1]

egen ordering = rank(random_number), unique

gen treatment = “”

*Control treatment

replace treatment = “C” if ordering <= _N/3

*Standard treatment

replace treatment = “TS” if ordering <= 2*_N/3 & ordering > _N/3

*Herd treatment

replace treatment = “TH” if ordering <= 3*_N/3 & ordering > 2*_N/3

**Appendix C: Power calculations**

The implementation of our randomized controlled trial was guided by an aim to run the study among the entire elderly population (aged 65 years and above) in two different samples. The randomization took place at the household (cluster) level to avoid sending letters with different contents to same household members. These two practical design principles determine our total sample size and the number of clusters (households) in our samples. Using information on sample sizes and number of clusters, we report the minimum detectable effect size (MDE) for different treatment effects and assess whether our null findings identify the absence of a true effect or signify a lack of statistical power.

The MDE is a metric to measure the smallest effect that would have been detectable given our sample sizes and clusters. Here we compute the MDEs with α = 0.05 and 0.80 power using different intracluster correlations for each pairwise comparison of our treatments. We note that the practice of reporting MDEs is substantially more conservative than simply stating the bounds of the 95% confidence interval.

*
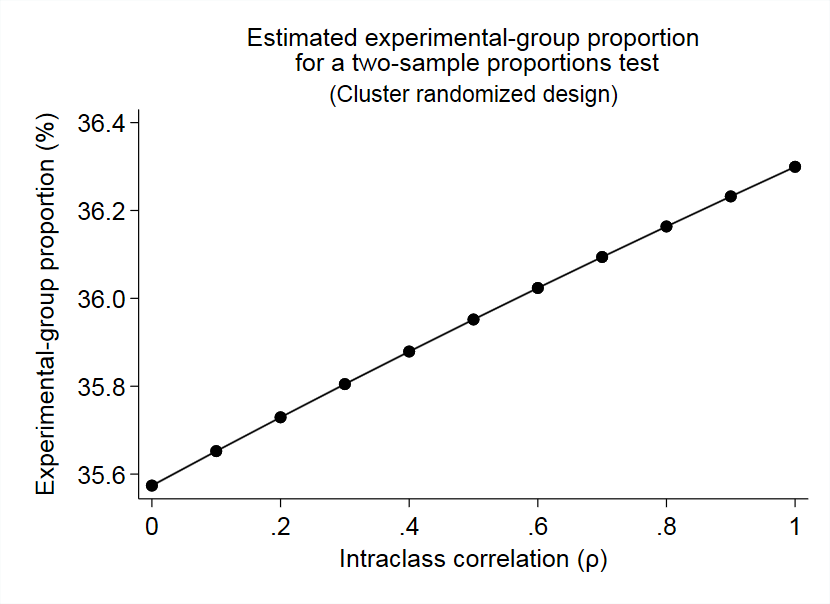
*

**Fig A:** Minimal detectable effect sizes (with α = 0.05 and 0.80) for treatment comparisons by intracluster correlation coefficients in the Western region

*Western region – Treatment comparisons:*

Our analysis sample includes 7324 (5221 clusters) individuals that are randomized into three different treatments: Control treatment (N = 2450, 1732 clusters), Individual-benefit treatment (N = 2445, 1738 clusters) and Individual-and-social-benefit treatment (N = 2429, 1751 clusters). Using a baseline immunization rate of 31.8% and assuming 80% power for a 5% (two-sided) level test, we determine that the (average) sample size of 2441 individuals and the average of 1740 clusters per treatment yields a minimal detectable effect size that varies from 3.8 percentage points to 4.5 percentage points depending on the intraclass correlation within clusters (Fig A in S1 Appendix).

*Western region – The effect of any type of reminder:*

Our analysis sample for estimating the impact of a reminder *per se* includes 7324 (5221 clusters) individuals that are divided into two groups: (i) Any type of reminder (N =4874, 3489 clusters) and (ii) Control treatment – no reminder (N = 2450, 1732 clusters). Using a baseline immunization rate of 31.8% and assuming 80% power for a 5% (two-sided) level test, we determine that this comparison yields a minimal detectable effect size that varies from 3.3 percentage points to 3.9 percentage points depending on the intraclass correlation within clusters (Fig B in S1 Appendix).


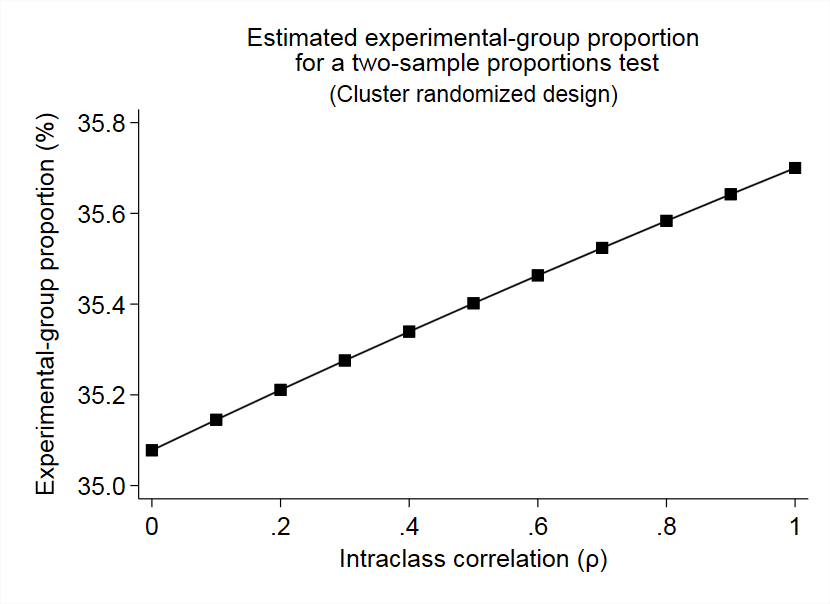


**Fig B:** Minimal detectable effect sizes (with α = 0.05 and 0.80) for the joint effect of any type of reminder by intracluster correlation coefficients in the Western region

*Southern region – Treatment comparison:*

Our analysis sample in the Southern region includes 40271 (29395 clusters) individuals that are randomized into two different treatments: Individual-benefit treatment (N = 19996, 14571 clusters) and Individual-and-social-benefit treatment (N = 20275, 14824 clusters). Using a baseline immunization rate of 57.7% and assuming 80% power for a 5% (two-sided) level test, we determine that this treatment comparison yields a minimal detectable effect size that varies from 1.4 percentage points to 1.6 percentage points depending on the intraclass correlation within clusters (Fig C in S1 Appendix).


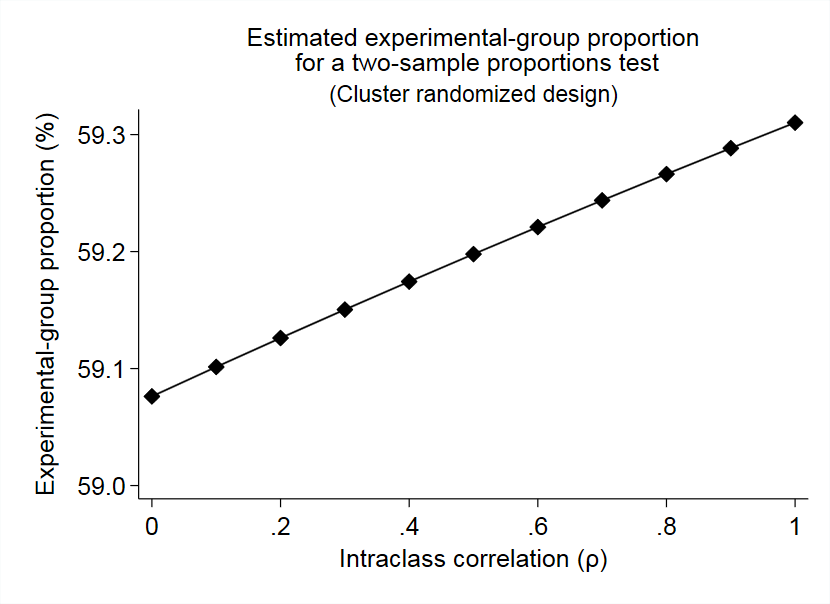


**Fig C:** Minimal detectable effect sizes (with α = 0.05 and 0.80) for the treatment comparison by intracluster correlation coefficients in the Southern region

**Appendix D: Supplementary Figures and Tables**

**D1: Robustness**

| **Table A**: Average treatment effects estimated using linear probability models, logit models and generalized mixed effects regression with random effects | | | | |
| --- | --- | --- | --- | --- |
| Western Region | | | Southern Region | |
| Diff. Any type of reminder  – No reminder | | Diff. Treatment I  – Treatment I + S | Diff. Any type of reminder  – No reminder | Diff. Treatment I  – Treatment I + S |
| Linear probability model | | | | |
| 6.4 pp | | 2.9 pp | - | - 0.2 pp |
| [3.6 pp – 9.1 pp] | | [0.0 pp – 6.1 pp] | - | [-1.3 pp – 0.9 pp] |
| (P < 0.001) | | (P = 0.087) | - | (P = 0.724) |
| Logit – Marginal effects | | | | |
| 6.4 pp | | 2.9 pp | - | - 0.2 pp |
| [3.6 pp – 9.1 pp] | | [0.0 pp – 6.1 pp] | - | [-1.3 pp – 0.9 pp] |
| (P < 0.001) | | (P = 0.087) | - | (P = 0.724) |
| Random effects model | | | | |
| 6.3 pp | 2.6 pp | | - | - 0.1 pp |
| [3.7 pp – 9.0 pp] | [-0.1 pp – 5.7 pp] | | - | [-1.2 pp – 1.0 pp] |
| (P < 0.001) | (P = 0.107) | | - | (P = 0.840) |
| Notes: This table reports differences between treatments. Squared brackets show 95% confidence intervals. P-values refer to the test of difference between treatments. To take into account the clustered randomization design linear probability models and logit models use standard errors clustered at household level, random effect model includes household as a random intercept. | | | | |

| **Table B**: Average treatment effects with and without control variables | | | | |
| --- | --- | --- | --- | --- |
| Western Region | | | Southern Region | |
| Diff. Any type of reminder  – No reminder | | Diff. Treatment I  – Treatment I + S | Diff. Any type of reminder  – No reminder | Diff. Treatment I  – Treatment I + S |
| **Panel A:** No control variables | | | | |
| 6.4 pp | | 2.9 pp | - | - 0.2 pp |
| [3.6 pp – 9.1 pp] | | [0.0 pp – 6.1 pp] | - | [-1.3 pp – 0.9 pp] |
| (P < 0.001) | | (P = 0.087) | - | (P = 0.724) |
| **Panel B:** With control variable for prior immunization history (influenza vaccine in 2017-2018) | | | | |
| 6.7 pp | | 0.2 pp | - | - 0.4 pp |
| [4.8 pp – 8.6 pp] | | [-0.2 pp – 2.5 pp] | - | [-1.3 pp – 0.4 pp] |
| (P < 0.001) | | (P = 0.896) | - | (P = 0.355) |
| **Panel C:** With control variables for prior immunization history (influenza vaccine in 2017-2018) and demographics (age, gender, household size) | | | | |
| 6.8 pp | 0.1 pp | | - | - 0.4 pp |
| [4.9 pp – 8.7 pp] | [-2.3 pp – 2.5 pp] | | - | [-1.2 pp – 0.5 pp] |
| (P < 0.001) | (P = 0.942) | | - | (P = 0.370) |
| Notes: This table reports average treatment effects with and without control variables. Panel A does not include any control variables. Panel B includes a control variable for prior immunization history (influenza vaccination status during the previous influenza season). Panel C includes a control variable for prior immunization history (influenza vaccination status during the previous influenza season) and control variables for demographics (age, gender and household size) Squared brackets show 95% confidence intervals. P-values refer to the test of difference between treatments. All standard errors are clustered at household level. | | | | |

| **Table C:** The effect of reminders on influenza vaccine coverage by prior immunization history – Random effects linear model | | | | | | |
| --- | --- | --- | --- | --- | --- | --- |
|  | Influenza vaccine coverage | | | | | |
|  | Conditional on  influenza vaccination  2017 – 2018 | | Conditional on  influenza vaccination 2011-2018 | | Conditional on  any vaccination  2011-2018 | |
|  | (1)  Vac. | (2)  Unvac. | (3)  Vac. | (4)  Unvac. | (5)  Vac. | (6)  Unvac. |
| Regression Coef:  Effect of any reminder (vs. no reminder) | 0.021  (.017) | 0.091***  (.012) | 0.052**  (.019) | 0.088***  (.012) | 0.060***  (.016) | 0.053***  (.014) |
| Observations | 2196 | 5128 | 3243 | 4081 | 5308 | 2016 |
| Coverage in control group (%) | 87.3% | 10.7% | 68.0% | 6.3% | 45.5% | 5.0% |
| Notes: Reported regression coefficients are derived using random effects linear models that allow cluster level heterogeneity (random effects) at the household level. All regressions are estimated at the individual level. Parentheses present standard errors from the random effects models. *** p < 0.01, ** p < 0.05, * p < 0.1. | | | | | | |

**D2: Cross-vaccine spillovers**

We examined whether receiving a reminder for influenza vaccination increases vaccine coverage for other common vaccines among our target population group. These types of cross-vaccination spillovers may occur through several behavioral channels. First, the reminders could increase the interaction between citizens and health care personnel who administer vaccinations. This interaction may lead to information exchange where providers inform citizens about the possibility of receiving other age-appropriate vaccinations. Second, the reminders may encourage individuals to gather knowledge about other available age-appropriate vaccinations from other available information sources (e.g. internet, books, and brochures). Third, the reminders may lead to changes in perceived confidence in vaccination in general and alter the inclination to take available age-appropriate vaccines.

We utilize the fact that our data included comprehensive patient records about all vaccines received after the implementation of the experiment. We estimate cross-vaccination spillovers in the Western region separately for pneumococcal conjugate vaccine (PCV), diphtheria vaccine (DV) and tick-borne encephalitis vaccine (TBE).

Table E in S1 Appendix presents the cross-vaccination spillovers. The first column shows the effect on PCV, second column on DT, third column on TBE vaccination. The fourth column shows the effect of having received any of the three vaccinations. There were no cross-vaccination spillovers. Using the 95% confidence intervals, we can rule out for PCV, DT, and TBE effects smaller than 0.5 percentage points and larger than 0.7 percentage points. For any vaccine, we can rule out effects smaller than -1.1 percentage points and larger than 0.9 percentage points. Overall, we find that influenza vaccination reminders do not cause any cross-vaccination spillovers.

| **Table D:** Cross-vaccination spillovers to other age-appropriate vaccines | | | | |
| --- | --- | --- | --- | --- |
|  | PCV | DT | TBE | Any vaccine |
|  | (1) | (2) | (3) | (4) |
| Regression Coef:  Effect of any reminder (vs. no reminder) | 0.001  (.004) | 0.001  (.003) | 0.001  (.001) | -0.001  (.005) |
| Sample mean (%) | 0.5% | 1.6% | 0.2% | 3.9% |
| Observations | 7.324 | 7.324 | 7.324 | 7.324 |
| Notes: Reported regression coefficients are derived using linear probability models. All regressions are estimated at the individual level. Standard errors in parentheses are clustered at the household level. *** p < 0.01, ** p < 0.05, * p < 0.1. | | | | |
