# Supplementary material for: Information nudges for influenza vaccination: Evidence from a large-scale cluster-randomized controlled trial in Finland
Source: PLoS Med. 2022 Feb 9;19(2):e1003919. doi: 10.1371/journal.pmed.1003919 (PMC8870595; doi:10.1371/journal.pmed.1003919)
Supplement: S1 Protocol — (DOCX) [file pmed.1003919.s003.docx]

Information nudges for influenza vaccination: Evidence from a large-scale cluster-randomized controlled trial

*Research plan / study protocol*

Lauri Sääksvuori, Cornelia Betsch, Hanna Nohynek,

Heini Salo, Jonas Sivelä, Robert Böhm

*1. Introduction*

Vaccination is often considered to be one of the most effective and successful means of preventive medicine (WHO, 2013). However, increasing levels of vaccine hesitancy pose a serious threat to the success of various vaccination programs (MacDonald, 2015 and Schmid et al., 2017). A prominent example of vaccination hesitancy is vaccination against seasonal influenza. Even though influenza is estimated to lead to 3 to 5 million cases of severe illness and 250.000 to 500.000 deaths every year (WHO, 2014), low influenza vaccine coverage within specific risk groups remains a challenge throughout the globe (WHO, 2015). In many countries, vaccinating against seasonal influenza is recommenced for all individuals aged 65 and above and vaccines are offered free of charge to all individuals in this age group. Despite the recommendation and low individual cost of vaccinating, the rates of influence vaccinations constantly remain below the socially optimal levels in the target populations.

Low rates of vaccination coverage put both the vaccine refusers and public health at risk. The herd immunity threshold required to indirectly protect unvaccinated individuals due to reduced pathogen spreading in the population is in many cases not reached (Fine et al., 2011). However, with increasing vaccination rates, herd immunity may effectively prevent the infection of people who are not able to get vaccinated themselves because, for instance, they are too young or immunocompromised. Considering the positive effect of one’s own vaccination on others, vaccination is also an act of pro-sociality (Betsch et al., 2013 and Betsch et al., 2017)

The aim of proposed research is to test the effectiveness of communicating the concept of herd immunity on actual influenza vaccine coverage in a naturally-occurring setting using a randomized controlled trial.

*2. Conceptual framework*

Using behavioural insights to increase individual vaccine uptake has received increasing attention and is considered to be a promising tool to increase vaccination uptake (Betsch et al. 2015). Here, we propose to test the effectiveness of a novel behavioural intervention building on the communication of herd immunity. Previous laboratory and online experiments have shown that communicating the concept of herd immunity increases individuals’ (hypothetical) intention to get vaccinated (Betsch et al., 2013, Betsch et al., 2017 and Böhm et al., 2018). The positive effect of communicating the concept of herd immunity is particularly pronounced when (*i*) the vaccination uptake rate is low (vs. high), (*ii*) the individual vaccination costs are low (vs. high), (*iii*) the social benefit (vs. the individual benefit) of herd immunity is emphasized, (*iv*) the beneficiaries of herd immunity are not able (vs. not willing) to get vaccinated themselves, and (*v*) the communication is based on an interactive simulation (vs. text-based explanation). However, despite the accumulating evidence regarding the effectiveness and boundary conditions of communicating herd immunity on vaccination attitudes, previous research has exclusively relied on hypothetical vaccination decisions under highly controlled conditions.

The aim of the present research is to test the effectiveness of communicating the concept of herd immunity on actual influenza vaccine coverage in a naturally-occurring setting using a randomized controlled trial. In addition to a control condition (no information about vaccination campaign against seasonal influenza), we propose two treatments that employ different strategies to inform citizens about the benefits of influenza vaccines. The treatment containing information about the herd immunity is based on previous studies of hypothetical vaccination decisions.

We are going to conduct a randomized controlled trial among older adult population in Finland. The National Institute for Health and Welfare (PIs Sääksvuori and Nohynek) will administer and conduct the studies. Participants will be Finnish individuals across several municipalities for whom influenza vaccination is recommended (aged 65 years and above).

*3. Experimental design*

We run a large-scale natural field experiment to understand the behavioural processes that guide decisions to vaccinate and test alternative strategies to communicate the concept of herd immunity. The experiment varies the text of postal letters send to individuals eligible for free influenza vaccines. The experiment is run in collaboration with the municipal health authorities. The municipalities that have confirmed their participation in the study are: Espoo, Maalahti, Korsnäs, Närpiö, Kaskinen ja Kristiinankaupunki.

We implement the randomized study in municipalities with varying historical vaccination uptake rates (historical and current vaccination coverage based on the Finnish vaccination register). Implementation in different municipalities with different current vaccination rates will give further information about the generalizability of the findings in different cultural and socio-economic environments and may enable us to test the average indirect effect of the treatments (Spillover effect, see section 4.5)

We identify using the Population Register all individuals who are born in year 1953 or before and resided in the participating municipality on July 01, 2018. We will obtain the postal addresses of these individuals using the Population Register and randomly assign individuals to treatment conditions that differ in the wording of the information material. Note that we will send the same letter separately to both individuals residing in the same address to avoid the situation that same persons living in the same household receive letters with different wordings. Importantly, the extract from the Population Register will also include an individual identifier (social security number) that can be used to match the received type of letter (treatment) with the actual vaccination decision recorded in the Care Register for Health Care to objectively evaluate the impact of received information on vaccination coverage. The matching of postal addresses and vaccination data will be conducted using pseudonymized identifiers. The pseudonymization of individual identifiers will be performed separately for both datasets by professional data management and data security staff of the National Institute for Health and Welfare. Consequently, the researchers conducting the data analysis will be able to access only pseudonymous data without a possibility to directly identify individuals from the data set. The data will be stored according to the data protection legislation and regulations of the National Institute for Health and Welfare during the research project.

We randomly assign individuals to treatment conditions that differ in the wording of the letters (attached). The authority’s standard letter serves as the baseline condition (T1). This letter is extended by communicating the concept of *herd immunity* and appealing to *pro-social preferences* by highlighting the fact that vaccinations may effectively prevent infections among people who are not able to vaccinate themselves (especially babies) (T2). To summarize, our study includes a control treatment (T0, no letter) and two treatment arms with the following contents: T1 – Standard letter, T2 – Herd immunity letter.

*4. Analysis Plan*

*4.1 Unit of Analysis*

Our primary outcome variable is individual level vaccination decision. In addition, we estimate average treatment effects by municipality and test whether different treatment arms have uniform effects across municipalities that differ in their baseline vaccination coverage.

*4.2 Average treatment effects*

We run the following linear probability model to estimate the average treatments effects:

$Y_{i}=\alpha+\beta_{1}Mailing+\beta_{2}Herd+\varepsilon_{i}$,

where $Y_{i}$ indicates whether individual *i* has received an influenza vaccine by the end of influenza season 2018 - 2019. Variable *Mailing* indicates whether individual *i* is in a mailing group or in the control group receiving no mailings. Variable *Herd* is a treatment dummies indicating whether individual *i* belongs to a treatment arm with herd immunity information. We cluster standard errors at the household level to account for correlation at the household level.

In addition to our primary interest on the overall effectiveness of different treatment arms, we investigate the effect of mailings by municipalities that vary in their baseline vaccination rates and estimate the following equation using linear probability model:

$Y_{i,t}=\alpha+\beta_{1}{Mailing}_{t}+\beta_{2}{Baseline}_{t-1}+\beta_{3}Mailing*{Baseline}_{t-1}+\varepsilon_{i,t}$,

where variable *Baseline* denotes the vaccination coverage by municipality in the previous year (2017). Thus, $\beta_{3}$ shows the differential average treatments effect of mailing by municipality.

We run all estimations primarily using linear probability models to improve comparability between estimations as estimating equations with interaction terms and computing correct interaction effects in non-linear models may require tedious computing (Ai and Norton, 2003.

*4.3 Statistical power*

We present in the following some standard power estimates to assess the minimum detectable effect size. Figure 1 shows the minimum detectable (absolute) effect size between two treatments as a function of sample size with test power .80 and .90. Power calculations are based on treatment comparisons using linear probability model. Here we assume that alpha = 0.05 (two-sided). Figure 2 shows the minimum total sample size needed to detect a standardized effect size varying from 0.05 to 0.25 (assuming test power .80 and alpha 0.05). Note that the power calculations do not account for potential corrections for multiple hypotheses testing or clustering of standard errors to account for potential correlation of standard errors.


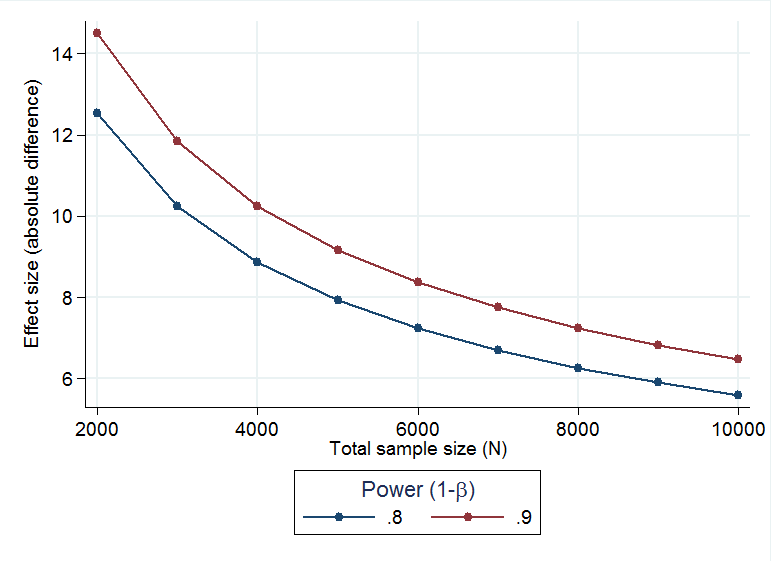


Figure 1. Minimum detectable effect size as a function of sample size and statistical power.


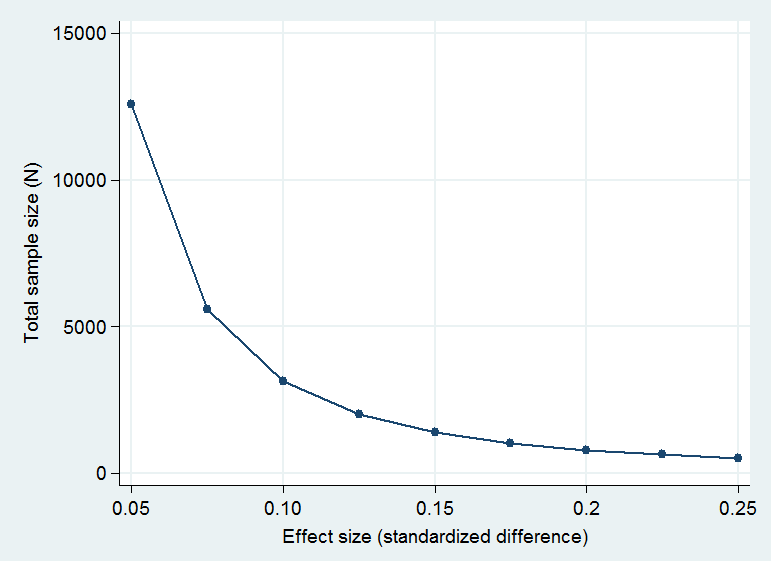


Figure 2. Minimum total sample size needed to detect a standardized effect size varying from 0.05 to 0.25 (assuming test power .80 and alpha 0.05).

*4.4 Attrition*

We do not expect significant attrition. The expected main sources of attrition are migration from the municipality and mortality. Migration from the target municipality to abroad during study period is expected to be less than 5 percent. We expect that attrition is not correlated with the treatment status.

*4.5 Spillover effects*

Our RCT is based on household-level randomization. The institutional context, the large coverage of target groups and relatively short study period (from the reception of letter until the end influenza season) are likely to substantially limit potential spillover effects between the treatment arms and control group. We design the implementation of various treatment arms such that two (or more) individuals over 65-years of age living in the same household will be assigned to same treatment arm. This practice eliminates potential spillover effects between individuals living in the same household.

The main concern related to potential spillover effects pertains to the treatment comparison between non-treated individuals and treated individuals (in any treatment arm). It is important to note that our main outcome is vaccination coverage which is unlikely to cause substantial externalities and affect the *vaccination coverage* of individuals who do not receive any letter informing the recipient about the upcoming influenza season. Likewise, there is no room for general equilibrium effects where (expected) increase in vaccination coverage in treatment groups would affect the supply of vaccines. The most notable potential channels of spillover effects between control and treatments groups operate through social interaction between the non-treated and treated individuals. For example, individuals who receive letters may share their information about the herd effect with non-recipients. It is difficult to ex ante assess if this kind of spillovers may lead us to underestimate the true effect of the mailings.

Despite the limited size of potential spillover effects, our experimental design where we assign individuals into treatments in several municipalities may enable us to measure the effect of treatments also on the non-treated individuals. There are three distinct types of individuals defined by individual treatment status and place of residence: (i) Treated individuals in the treated municipalities receiving mailing, (ii) non-treated individuals in treated municipalities who do not receive mailings, but may be indirectly affected by the treatment, (iii) non-treated individuals in non-treated municipalities who are not affected (assuming that the potential spillover effects occur only within treated municipalities). Thus, the presence of these three types of individuals enables us to estimate the Average Treatment Effect (ATE) and the average Indirect Treatment Effect (ITE).

$$ATE=E\left( Y_{1}-Y_{0} \right|T=1, M=1)=E\left( Y | T=1,M=1 \right)-E(Y|T=0,M=1)$$

$ITE=E(Y_{1}-Y_{0}\left| T=0,M=1 \right)=E\left( Y | T=0,M=1 \right)-E(Y|T=0,M=0)$,

where Y is the outcome (Decision to vaccinate), T is individual treatment status and M is an identifier for the municipalities sending mailings to their citizens. However, note that we estimate the ITE accurately if spillover effects occur only within the treated municipalities.

*5. Data Management*

We will follow the general Finnish guidelines on research ethics for research data management (for a more detailed description of data management see the separate data management plan). In addition, we will follow the data availability policy applied by targeted journals.

The linked data sets will be made available to all interested researchers during the whole research period given that the researchers have acquired appropriate data permissions according to Finnish data protection legislation. The data permission procedures will be handled by the professional staff in charge of the data management and security at the Finnish National Institute for Health and Welfare. Since the register databases include sensitive individual-level information, only pseudonymized merged dataset without direct identifiers will be made available to interested researchers.

**References**

Ai, C., & Norton, E. C. (2003). Interaction terms in logit and probit models. *Economics letters*, 80(1), 123-129.

Betsch C, Böhm R, Korn L. Inviting free-riders or appealing to prosocial behavior? Game-theoretical reflections on communicating herd immunity in vaccine advocacy. *Health Psychol*. 2013; 32(9):978-85. doi:10.1037/a0031590.

Betsch C, Böhm R, Chapman GB. Using Behavioral Insights to Increase Vaccination Policy Effectiveness. *Fiske ST, ed. Policy Insights from Behav Brain Sci*. 2015;2(1):61-73.

Betsch C, Böhm R, Korn L, Holtmann C. On the benefits of explaining herd immunity in vaccine advocacy. *Nat Hum Behav*. 2017;1(3):56. doi:10.1038/s41562-017-0056.

Böhm R, Betsch C, Korn L. Selfish-rational non-vaccination: Experimental evidence from an interactive vaccination game. *J Econ Behav Organ*.

Böhm R, Meier NW, Korn L, Betsch C. Prosocial vaccination to protect (un)intentional non-vaccinators.; 2018.

Fine P, Eames K, Heymann DL. Herd Immunity: A Rough Guide. *Clin Infect Dis*. 2011;52(7):911-916. doi:10.1093/cid/cir007.

MacDonald NE, SAGE Working Group on Vaccine Hesitancy. Vaccine hesitancy: Definition, scope and determinants. *Vaccine,* 2015;33(34):4161-4164.

Schmid P, Rauber D, Betsch C, Lidolt G, Denker M-L. Barriers of Influenza Vaccination Intention and Behavior – A Systematic Review of Influenza Vaccine Hesitancy, 2005 – 2016. Cowling BJ, ed. *PLoS One.* 2017;12(1):e0170550.

WHO. Global vaccine action plan 2011-2020. Geneva: WHO Press; 2013.

WHO. Seasonal influenza. Fact sheet. 2014.

WHO. The global action plan for influenza vaccines report of the tenth meeting of the advisory group of the WHO global action plan for influenza vaccines. 2015.
