# Supplementary material for: Information nudges for influenza vaccination: Evidence from a large-scale cluster-randomized controlled trial in Finland
Source: PLoS Med. 2022 Feb 9;19(2):e1003919. doi: 10.1371/journal.pmed.1003919 (PMC8870595; doi:10.1371/journal.pmed.1003919)
Supplement: Attachment — Submitted filename: PLOS_MED_R&R_D-20-02666R1_Ref_3_response_letter.docx [file pmed.1003919.s004.docx]

Dear Reviewer,

We would like to thank you for your detailed report and constructive comments that have helped us improve the quality of our manuscript. We aspire to address all your concerns and comments in the revised version of our manuscript. Please find a detailed point-by-point response to your comments below. Your comments are shown in bold font. Our responses are written using regular font.

**________________________**

***** Reviewer #3:**

**The authors present the results of an interesting pragmatic trial of the effect of letters, including standard information or information about herd immunity/social emphasis) versus no letter across two geographic regions in Finland. A particular strength of this study is the careful selection of two different regions with different prior vaccination coverage to enhance generalizability and explore the effect of letters among patients with different prior exposure to the vaccine. Another strength is the long duration of individuals included in the study (>9 years) and complete coverage of influenza vaccination outcomes through population-level claims. The results largely confirm a large prior trial conducted in the US (along with other trials of mailed letters) that show that mailed letters can increase vaccination rates and that additional tailoring does not provide massive additional better compared with no letter.**

**Major comments**

**- Tailored letter: The authors emphasize that the letter is "tailored" about social benefits of vaccination due to herd effect. I posit instead that the letter emphasizes social consequences rather than just informational; it is not exactly tailored to individuals, so the language is inaccurate. It is also inconsistent, as the letters are described differently in the abstract versus the body of the manuscript. The authors should stick with what is written in the manuscript, as that appears more accurate.**

We thank you for mentioning that our individual-and-social-benefit reminder was not really “tailored” to individuals. We agree that describing this letter reminder as a tailored letter was potentially misleading and do not use the term “tailored” in the revised manuscript. To be more consistent with the terminology, we use throughout the paper term “individual-and-social-benefit reminder” to describe the letter that contained information about the additional social benefits of vaccination. Please note that we now also label our treatments as “reminders” instead of “letters” to be consistent with the previous literature (as suggested by the reviewer #2).

**- No control group in one region: The authors did not have a control group in the southern region (the region with better vaccination uptake). This means that the control group is likely biased towards being worse than the intervention group already (and therefore makes the overall results less believeable), because the only controls were in the region where there was worse baseline vaccination coverage. The authors recognize this as a limitation but need to be more careful in their interpretation, especially against control. It is understandable why no letter was sent, but the authors need to present results more clearly (including in the abstract) for just the Western region separately to overcome this.**

It is correct that we did not have a control group (receiving no reminder) in the Southern region. This is an obvious limitation of the study. However, we want to clarify that we do not compare the vaccination coverage in the treatment arms in the Southern region against the (no letter) control group in the Western region exactly for the reason that the regions have very different population-wide vaccination coverage at baseline. We think that this limitation mainly relates to the generalizability of our findings from populations with low baseline vaccination coverage to populations with high baseline vaccination coverage. To clarify this point, we acknowledge this limitation and aim to present results more clearly in the revised manuscript. To make it explicit that the increase in vaccination coverage was only observed in the low-coverage region we have made the following revisions to the manuscript:

(1) We write in the abstract (when referring to the results from the Western region) that these effects were observed “in the region with low vaccination coverage” and report the difference between the individual-benefit reminder and the individual-and-social-benefit reminder separately for each region.

(2) We write in the abstract that “Sending reminders is an effective and easily scalable low-cost intervention strategy to increase vaccine uptake in an elderly population with low vaccination coverage.”

(3) We write in the Discussion that “We observed that a low-cost and scalable intervention relying on individually mailed letters substantially increases influenza vaccination coverage in a population with low baseline vaccination coverage.”

**Minor comments**

**-Abstract (Findings): The authors state that individually mailed letters increased influenza vaccination coverage, but it is not clear which arm (treatment) the results refer to, nor the comparison. This should be clarified. The results for all 3 arms should be presented, rather than subgroups, which is what is being presented now. The results across arms is shown in the last sentence, but the results should be presented independently.**

Thank you for pointing us to this ambiguity in the abstract. We have rewritten the abstract to make clear (i) which region received which treatments and (ii) which results relate to which treatment comparison and which sample population. Accordingly, we also present the comparison between the individual-benefit and the individual-and-social-benefit reminder separately for each sample population.

**- Statistical analysis (Power): The authors state that they assume an ICC of 0.5; is there citation for this or baseline data to support?**

Thank you for asking to clarify the reasoning behind this assumption. To address the concerns raised by reviewer #1, we have rerun all MDE calculations using the observed baseline vaccination coverage and clearly state that we are calculating the MDE. Consequently, we now draw our inferences about the MDE using observed vaccination coverage and observed ICC of 0.7

**-Table 1: The authors should provide some data (e.g., absolute standardized differences or p-values) to describe balance in the groups. There is some evidence of imbalance across the arms on for example influenza vaccination receipt in prior season within region across arms, which biases towards seeing an effect in the letters (e.g., 33.5% vs. 32.1%).**

Thank you for this comment and suggestion. We provide as part of the revised submission a new table that includes a description of balance checks (absolute standardized differences and p-values) across treatments and available covariates. Using a critical statistical-significance threshold of p < 0.05, we find one statistically significant difference in covariate balance across treatments and included covariates (influenza vaccination receipt in prior season was higher in Treatment I than in Treatment I + S in the Western region). We note that this number of statistically significant imbalances is expected to arise by chance alone. This single statistically significant imbalance does not invalidate our randomization procedure or the data. To complete the picture, we provide results from a joint test of significance across all available covariates and run a following regression using all available (six) covariates: Treatment indicator = α + β1*X1 + β2*X6 + … β5*X6 + ε and test the joint hypothesis that β1 = β2 =… β3 = 0). Using this joint test of orthogonality, we find that there are no systematic imbalances between the treatment arms.

**- Control variables: The authors state that they did not include any control variables in the analysis but should adjust for imbalanced covariates in at least supplementary analyses.**

Thank you for the comment and suggestion. We provide as part of the revised submission new robustness analyses that include different sets of control variables. The more parsimonious models include a control variable for prior immunization history (as suggested by the balance checks), whereas the less parsimonious models include control variables for prior immunization history and demographics (age, gender, household size).

We find that including the above-mentioned control variables mainly lead to smaller standard errors, as expected, because control variables absorb the effects of imbalanced covariates. However, as suggested by the review report, including a control variable for prior immunization history also diminishes the point estimate for Treatment I vs. Treatment I + S comparison in the Western region. Overall, the inclusion of control variables supports our (prior) conclusion that communicating the social benefits of vaccination in addition to the individual benefit does not enhance influenza vaccine uptake.

**- Figure 2: The scale should be 0-100% to avoid overinterpretation.**

Thank you for this comment and suggestion. We have revised Figure 2 as suggested.

**Discretionary comments**

**- Abstract (Methods): The authors do not provide information about the modeling or statistical analysis plan in the abstract.**

Due to space limitations in the Abstract, we decided to stick to not reporting information regarding modeling or statistical analysis plan here.
