# Supplementary material for: Information nudges for influenza vaccination: Evidence from a large-scale cluster-randomized controlled trial in Finland
Source: PLoS Med. 2022 Feb 9;19(2):e1003919. doi: 10.1371/journal.pmed.1003919 (PMC8870595; doi:10.1371/journal.pmed.1003919)
Supplement: Attachment — Submitted filename: PLOS_MED_R&R_D-20-02666R2_Reviewer_#3_Nov_23_2021.docx [file pmed.1003919.s005.docx]

Dear Reviewer,

We would like to thank you again for your comments. They have helped us improve the quality of our manuscript. Please find a detailed point-by-point response to your critiques and remarks below. Your comments are shown in bold font. Our responses are written using regular font.

_______________

***** Reviewer #3:**

**Major comments:**

**1) Abstract: The abstract does not provide the referent group for the 6.4pp increase; is this compared with control or another type of letter? The authors state that individually mailed letters increased influenza vaccination coverage, but it is not clear which arm (treatment) the results refer to, nor the comparison. This should be clarified. The results for all 3 arms should be presented, rather than subgroups, which is what is being presented now. The results across arms is shown in the last sentence, but the results should be presented independently. In addition, some aspects of the abstract use words that could be over-interpreted, such as "crucially depends on", which is not supported by the data presented.**

Thank you for pointing out that the treatment arm and comparison group for the 6.4 pp increase was unclear. This is the effect of receiving any type of reminder letter (independent of the letter content) versus being in the control group without a reminder. We have now clarified this comparison and write in the abstract that: “The effect of receiving any type of reminder letter in comparison to control group (no reminder) was 6.4 percentage points (95% CI: 3.6 to 9.1, p < 0.001) in the region with low vaccination coverage.”

We agree that it is useful to present the results for all three treatment arms in the abstract. We now present the results from all treatment arms in both regions independently. We have revised the wording of conclusions in the abstract and deleted the expression “crucially depends on”. Following a very detailed suggestion by reviewer #2, we write in the conclusion that “the effectiveness of letter reminders about the benefits of vaccination to improve influenza vaccination coverage may depend on the prior vaccination history of the population.”

**Minor comments:**

**2) Randomization: Was 1:1 simple cluster randomization implemented or did it account for any blocking factors? This is not currently clear.**

We used simple 1:1 randomization without any blocking factors. We describe the randomization process in the revised manuscript and write that: “We used simple randomization without any blocking factors”.

**3) Outcome capture: Is there any chance that individuals could have gotten the flu shot outside of the 5-month follow-up period (particularly before)? How were these individuals treated in the analysis? It would likely bias towards the null but would be important to clarify.**

Thank you for asking to clarify if there was any chance that individuals could have gotten the flu shot outside of the 5-month follow-up. The Finnish National Vaccination Register contains records of all vaccinations given at public health care units in Finland since 2009. Using this database, we find that 10 individuals aged 65 years or above received a flu shot in the Western region between Jun 1, 2018 and Oct 17, 2018, and 210 individuals aged 65 years or above received a flu shot in the Southern region between Jun 1, 2018 and Oct 18, 2018. No flu shots were administered in these regions after March 18, 2019 (until the next influenza season). All individuals who received a flu shot between Jun 1, 2018 and Oct 17, 2018 have been excluded from the analyses. We now clearly state in the revised manuscript that individuals receiving a flu shot before the beginning of the follow-up have been excluded from the analyses and write that: “We excluded from the sample and statistical analysis also all individuals who received influenza vaccination before the beginning of the follow-up time. There were 10 individuals who received influence vaccination in the Western region between Jun 1, 2018 and Oct 17, 2018, and 210 individuals who received influenza vaccination in the Southern region between Jun 1, 2018 and Oct 17, 2018.”

**4) Households: Were any households more than 2-person? (e.g., siblings living with married siblings) The manuscript assumes only 2 adult persons/household.**

Thank you for asking to clarify if there were any households with more than 2-persons. The identification of households in our data is based on postal addresses (individuals with same postal address are interpreted as households, even though we are not able to verify that the persons form a household). Yes, there are cases in which more than 2-persons have the same postal address in our data. However, as we are not able to separate larger households (e.g. siblings living with married siblings) from nursing homes we have decided to exclude all housing units with more than two persons from the analysis sample.

The consequences of excluding housing units with more than 2-persons and individuals who received a flu shot between Jun 1, 2018 and Oct 17, 2018, are shown in Figure 1 (number of randomized households vs. number of analyzed households). We now clearly state in the revised manuscript that we excluded all housing units with more than 2-persons and write that: “However, individuals living in housing units with more than two persons (e.g., nursing homes) were excluded from the sample and statistical analyses after the randomization because these housing units could include private nursing homes that provide seasonal influenza vaccinations to all residents as part of their care plan.”

**5) Sample size calculations: It is not clear if the authors adjusted for multiple testing in the analysis (e.g., through Bonferroni). This is likely ok given their study question (and the null result) but would be good to clearly specify.**

Our computations of minimal detectable effects sizes do not account for potential corrections of multiple hypotheses testing. We now explicitly write in the main text (Section: Statistical analysis) that our sample size calculations do not adjust for multiple testing. “Our computations of minimal detectable effects sizes do not account for potential corrections of multiple hypotheses testing.”

**6) Table 1: The authors have not provided a description of balance changes across characteristics (e.g., ASDs or p-values) - this should be in Table 1, not online appendices. This was not sufficiently addressed in the prior revision.**

Thank you for mentioning again a need to provide a description of balance checks across characteristics. We agree that it is useful to report balance checks in the main text (instead of online appendices) and have now merged Table 1 with (previous) Table A5 that included ASD and p-values across all treatment comparisons. Moreover, we now shortly report the main findings from the balance tests in main text.

**7) Table 2: Please indicate which region these analyses are conducted in in the table. Also please include full titles of information to reduce the need for reviewers to go to footnotes.**

Thank you for asking to clarify the region of analyses and add information to the subheadings. We now mention the region in the table caption and table header. We now also clearly mention in the subheadings (under 1,2,3,4,5,6) whether the model is estimated in a sample of previously vaccinated vs. previously unvaccinated individuals.

**8) Abstract: "lack of no reminder treatment": clearer to say "no control"**

We agree and have changed the text as suggested.

**Discretionary comments:**

**9) "Text-based reminders" sounds like "text messaging" - recommend re-writing.**

We agree and have revised the sentence that included “text-based reminders”.

**10) It is confusing to abbreviate to "Treatment I" and "Treatment I+S" - recommend putting more in a technical appendix but not to use non-standard abbreviations**

Thank you for recommending to avoid using potentially confusing abbreviations. To address this recommendation, we have deleted all abbreviations “Treatment I” and “Treatment I+S” from the main text. We use treatment labels I and I+S only in figures and tables to save space and make them fit on page.
